# Dysmorphic Facial Features and Other Clinical Characteristics in Two Patients with* PEX1* Gene Mutations

**DOI:** 10.1155/2016/5175709

**Published:** 2016-11-02

**Authors:** Mehmet Gunduz, Ozlem Unal

**Affiliations:** Division of Metabolism and Nutrition, Ankara Children's Hematology-Oncology Research and Training Hospital, Ankara, Turkey

## Abstract

Peroxisomal disorders are a group of genetically heterogeneous metabolic diseases related to dysfunction of peroxisomes. Dysmorphic features, neurological abnormalities, and hepatic dysfunction can be presenting signs of peroxisomal disorders. Here we presented dysmorphic facial features and other clinical characteristics in two patients with* PEX1* gene mutation. Follow-up periods were 3.5 years and 1 year in the patients. Case I was one-year-old girl that presented with neurodevelopmental delay, hepatomegaly, bilateral hearing loss, and visual problems. Ophthalmologic examination suggested septooptic dysplasia. Cranial magnetic resonance imaging (MRI) showed nonspecific gliosis at subcortical and periventricular deep white matter. Case II was 2.5-year-old girl referred for investigation of global developmental delay and elevated liver enzymes. Ophthalmologic examination findings were consistent with bilateral nystagmus and retinitis pigmentosa. Cranial MRI was normal. Dysmorphic facial features including broad nasal root, low set ears, downward slanting eyes, downward slanting eyebrows, and epichantal folds were common findings in two patients. Molecular genetic analysis indicated homozygous novel IVS1-2A>G mutation in Case I and homozygous p.G843D (c.2528G>A) mutation in Case II in the* PEX1* gene. Clinical findings and developmental prognosis vary in* PEX1* gene mutation. Kabuki-like phenotype associated with liver pathology may indicate Zellweger spectrum disorders (ZSD).

## 1. Introduction

Peroxisomal disorders are a group of genetically heterogeneous metabolic diseases related to dysfunction of peroxisomes. Peroxisomes are mainly involved in lipid metabolism. They synthesise ether phospholipids, called plasmalogens, and beta oxidise very long chain fatty acids. They are also involved in oxidation of phytanic acid, formation of bile acids from mevalonate, and catabolism of lysine and glyoxylate [1]. Dysmorphic features, neurological abnormalities, and hepatic and gastrointestinal dysfunction can be presenting signs of peroxisomal disorders. Dysmorphic features may include craniofacial dysmorphism, skeletal abnormalities, shortened proximal limbs, calcific stippling of epiphyses, and renal cysts in different disorders linked to peroxisomal dysfunction [1]. Here we present dysmorphic facial features and other clinical characteristics in two patients with* PEX1* (Peroxisomal Biogenesis Factor 1) gene mutation [2].

## 2. Case I

**Figure 1:**
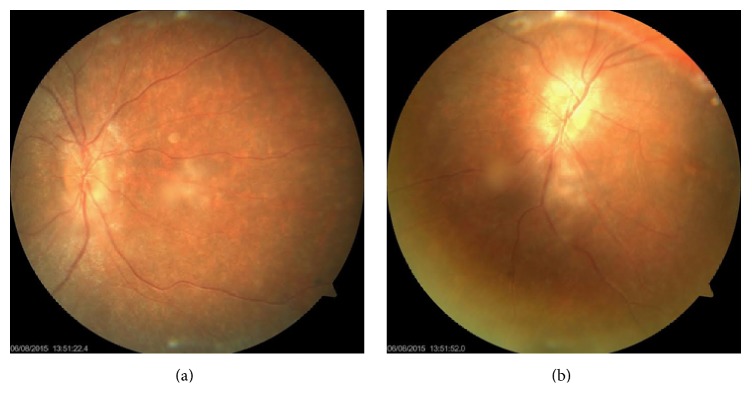
Appearance of pale optic disk and irregular optic disc margins.

**Figure 2:**
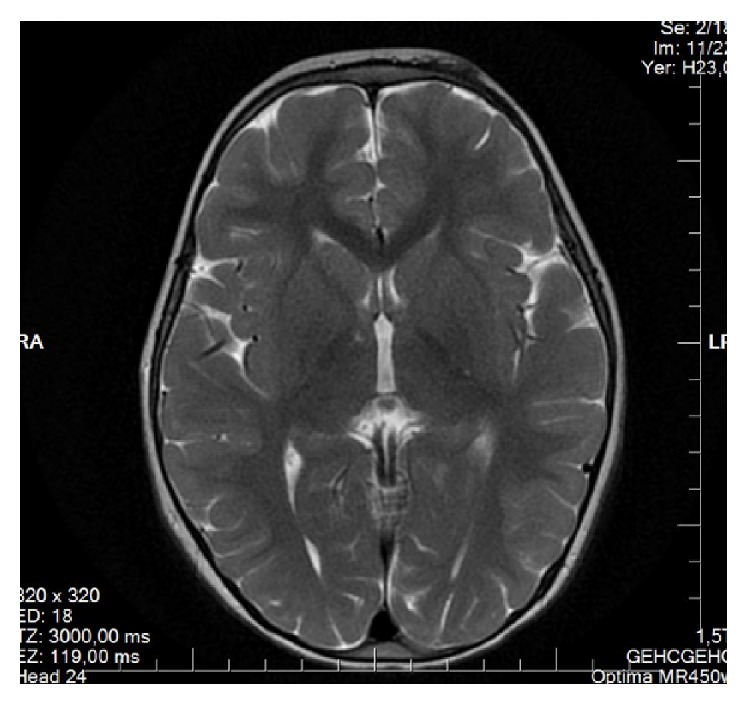
Nonspecific gliosis at subcortical and periventricular deep white matter.

One-year-old girl was referred to our center with neurodevelopmental delay, bilateral hearing loss, and visual problems. Parents were first cousins. The perinatal history was unremarkable. On physical examination, growth parameters were within normal limits. She had hearing loss and she was using bilateral hearing aids. Liver was palpable 6 cm below the right costal margin. Dysmorphic facial features including arched eyebrows, broad nasal root, low set ears, downward slanting eyes, epichantal folds, strabismus, and myopathic face were noticed. Laboratory findings revealed the following: ALT: 101 U/L (*N*: 0–37) and AST: 53 U/L (*N*: 0–41). Ophthalmologic examination suggested septooptic dysplasia. Optic disc was pale and optic disc margins were irregular (Figures 1(a) and 1(b)). Visual evoked potentials showed bilateral elongated P100 latency. Cranial magnetic resonance imaging (MRI) showed nonspecific gliosis at subcortical and periventricular deep white matter (Figure 2). Superior margin of the sella showed mildly upward convexity.

Very long chain fatty acid analysis revealed elevated C26:0, low C24:0, low C22:0, elevated C26:0/C22:0 ratio, highly elevated phytanic acid, and pristanic acid levels and was compatible with Zellweger spectrum (Table 1). Molecular genetic analysis for* PEX* genes indicated homozygous novel IVS1-2A>G mutation in the* PEX1* gene.

**Figure 3:**
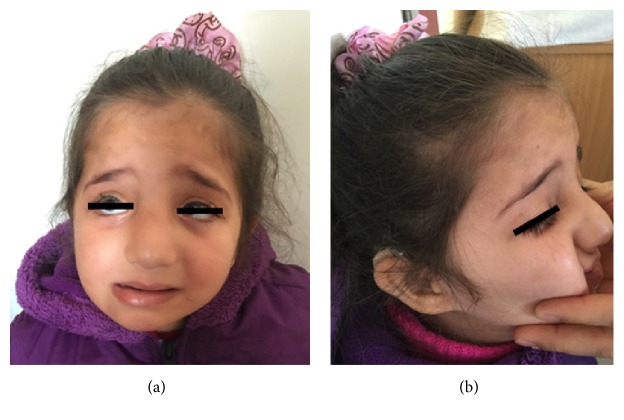
Dysmorphic facial features in Case I.

She is five years old now. She has delayed speech, cognitive delay, and prominent facial dysmorphic features (Figures 3(a) and 3(b)). She has no marked motor dysfunction.

## 3. Case II

**Figure 4:**
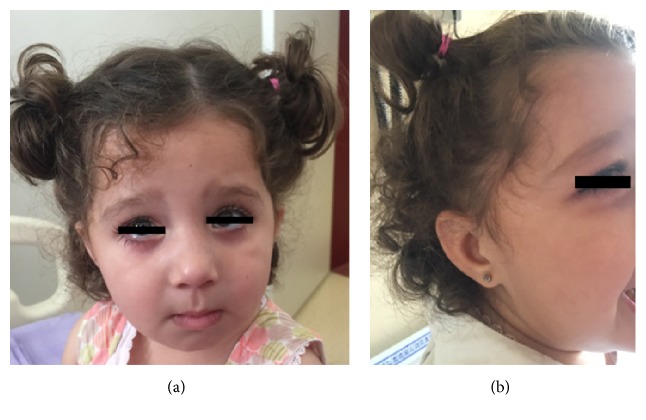
Dysmorphic facial features in Case II.

2.5-year-old girl was referred to our clinic for investigation of global developmental delay and elevated liver enzymes. She was born with an uneventful, term delivery. Her parents were first cousins. Mild hepatomegaly and elevated liver enzymes had been first detected incidentally when she was 3 months old. She could walk at 19 months, and she was talking with a few single words at 2,5 years. On physical examination, growth parameters were within normal limits. Liver was palpable 3 cm below the right costal margin. She had bilateral nystagmus. Dysmorphic facial features were determined similar to that defined in Case I, including arched eyebrows, broad nasal root, low set ears, downward slanting eyes, epichantal folds, strabismus, and myopathic face (Figures 4(a) and 4(b)).

Laboratory findings showed anemia, mildly elevated liver enzymes, and coagulopathy and revealed the following: WBC: 24.000/mm^3^, Hgb: 7.9 g/dL, Hct: 28.3%, MCV: 62,6 fL, RDW: 19.7%, plt: 533.000/mm^3^, AST: 51 U/L (0–35), ALT: 13 U/L (0–35), GGT: 24 U/L (4–22), APTT: 34.1 sec (19.7–27.6), PT: 14,7 sec (9.4–13.7), and INR: 1.26 (0.82–1.20).

Ophthalmologic examination findings were consistent with bilateral nystagmus and retinitis pigmentosa. Visual evoked potentials showed bilateral elongated P100 latency. Hearing assessment was normal. Echocardiography was normal except for thin patent ductus arteriosus. Abdominal ultrasonography showed mild hepatomegaly and increased liver parenchymal echogenicity. Doppler sonography of the portal venous system was normal. Cranial and diffusion weighted MRI were normal.

Very long chain fatty acid analysis revealed elevated C26:0, normal C24:0, low C22:0, elevated C26:0/C22:0 ratio, highly elevated phytanic acid, and pristanic acid levels and was compatible with Zellweger spectrum (Table 1). Molecular genetic analysis for* PEX* genes indicated homozygous p.G843D (c.2528G>A) mutation in the* PEX1* gene.

She is 3.5 years old now. She has moderate cognitive delay and mild motor delay. She can still talk with single words and she can climb the stairs by holding. She needed erythrocyte transfusions two times despite iron treatment in the follow-up period. At last admission her hemoglobin level decreased to 5 g/dL; anemia was consistent with iron deficiency. Leukocytes and platelet counts were normal.

## 4. Discussion

Peroxisomal disorders are a heterogeneous group of metabolic disorders that lead to impairment of peroxisome functions. Neurologic, hepatic, and gastrointestinal abnormalities are common clinical findings in different genetic abnormalities associated with peroxisomal dysfunctions. Dysmorphic features can have also prominent signs. Peroxisomal disorders are divided into two major categories including disorders of peroxisome biogenesis and deficiency of a single peroxisomal enzyme. The biogenesis of peroxisomes involves formation of peroxisomal membranes, import of peroxisomal membrane and matrix proteins, peroxisomal growth, division, and proliferation [3]. Peroxisome biogenesis disorders (PBD) are divided into two groups including Zellweger spectrum disorder (ZSD) and rhizomelic chondrodysplasia punctata type 1. Mutations in twelve different* PEX *genes that encode peroxins are responsible for PBD. Mutations in* PEX1 *gene are the most common cause of PBD [4]. Here we presented dysmorphic facial features and other clinical characteristics in two patients with* PEX1* gene mutation.

*PEX1 *gene encodes a member of the AAA ATPase family, a large group of ATPases associated with diverse cellular activities. The cytogenetic location is on chromosome 7q21-q22. This protein is cytoplasmic but is often anchored to a peroxisomal membrane where it forms a heteromeric complex and plays a role in the import of proteins into peroxisomes and peroxisome biogenesis. Mutations in this gene have been associated with complementation group 1 peroxisomal disorders such as neonatal adrenoleukodystrophy (NALD), infantile Refsum disease (IRD), classical Zellweger syndrome (ZWS), and ZSD [5]. There is clinical, biochemical, and genetic overlap among the three phenotypes, also known as ZSD. Clinical distinctions between the phenotypes are not sharply defined [6].

The broad clinical spectrum in PBD, single enzyme, and transporter deficiencies may be related to residual activity of the affected protein, genetic background, or environmental factors [7]. Dysmorphic features, neurologic abnormalities, ocular abnormalities, liver disease, renal defects, and patellar calcific stippling are the clinical characteristic findings of patients with classical ZWS. Survival is usually less than one year. Patients with atypical NALD and IRD may have similar clinical characteristics, but survival is longer [8]. Recently Braverman et al. recommended replacing these names with the overall classification of peroxisome biogenesis disorders in the ZSD [9]. Most of children with NALD die before three to five years of age. IRD is less severe than ZWS or NALD, most affected individuals can walk as in our patients, and many survive into adolescence. Some patients with peroxisome biogenesis disorders survive to adulthood. Particularly p.G843D mutation identified in our patient (Case 2) was reported in this group [6].

*PEX1* G843D allele detected in our patient (Case 2) has been associated with the less severe end of the phenotypical continuum of PBD, and peroxisomal matrix protein import was reported to be nearer to normal [4]. Poll-The et al. delineated the natural history of 31 PBD patients through systematic clinical and biochemical investigations. They excluded classical ZS and included all patients with a biochemically confirmed generalized peroxisomal disorder over 1 year of age. At the molecular level, 21 patients had mutations in the* PEX1* gene. The two most common* PEX1* mutations were the G843D (c.2528G>A) missense and the c.2097insT frameshift mutation. Patients having the G843D/G843D or the G843D/c.2097insT genotypes were compared. Patients homozygous for G843D generally had a better developmental outcome. However, they emphasized that next to the* PEX1 *genotype other factors determine the ultimate phenotype [6]. In our study Case 2 carrying this mild mutation had liver symptoms from 3-month-old, moderate cognitive delay and mild motor delay at 3.5 years old and dysmorphic features. She had no hearing impairment but ophthalmologic findings including nystagmus and retinitis pigmentosa. Severe persistent iron deficiency anemia was one of the striking findings in this patient.

Homozygous IVS1-2A>G mutation in the* PEX1* gene detected in Case I was novel. Her phenotype was also in ZSD consistent with IRD or peroxisome biogenesis disorders surviving to adulthood. She had bilateral hearing loss, delayed speech, cognitive delay, and prominent facial dysmorphic features. She had no marked motor dysfunction. Septooptic dysplasia was an interesting clinical finding in this patient.

Although most often patients with IRD have no obvious facial dysmorphia; PBD may be confused with other conditions owing to dysmorphic features, neurologic problems and vision and hearing impairment. Dysmorphic features including large fontanelles, a high forehead, epichantal folds, and abnormal ears may be mistaken for chromosomal disorders such as Down syndrome-like facies [10]. Ezgu et al. reported a case with late onset Zellweger syndrome who had some phenotypical findings which are also seen in Kabuki Syndrome. Arched eyebrows, large protuberant ears, blue sclerae, strabismus, epichantal folds, short nasal septum, myopathy, clinodactyly of the fifth fingers, and persistent fetal finger pads were dysmorphic features that they reported in their patient. They concluded that Zellweger syndrome should be included into the differential diagnosis of the patients with Kabuki-like phenotype and they emphasized abnormal liver functions [11]. Both of our patients had facial findings similar to patient reported by Ezgu et al. Kabuki-like dysmorphic facial features may be considered as a clinical finding of PBD.

Retinal dystrophy and optic nerve abnormalities are common in PBD-ZSD and may lead to another vision loss [1]. In our patients optic nerve abnormalities were prominent sign, but in Case 2, retinitis pigmentosa was detected. Visual evoked potentials were elongated in both patients. Hearing loss is also a common finding in patients with PBD-ZSD [1]. One of our patients needed bilateral hearing aids, but hearing assessment was normal in our other patient at 3,5 years old.

MRI is normal in some patients with IRD; no migration defects have been described in this least severe form of ZSD [7]. However, progressive leukoencephalopathy may occur following stable neurologic findings [9]. Corpus callosum, areas surrounding the lateral ventricles, internal capsules, peridentate white matter, brainstem, and central white matter of cerebellar hemispheres may be involved areas. In our patients, cranial MRI showed nonspecific gliosis at subcortical and periventricular deep white matter in Case I and cranial imaging was normal in Case II. The clinical course is variable in PBD [6]. Cognitive and motor development varies between severe handicap and moderate learning disorder with deafness and visual impairment related to retinopathy. Both of our patients had mild to moderate cognitive delay associated with visual problems and hearing impairment in one of our patients.

In conclusion, clinical findings, cranial imaging findings, and developmental prognosis are variable in* PEX1* gene mutation. Neuroimaging is not always correlated with neurologic outcome. Kabuki-like phenotype associated with liver pathology may indicate PBD-ZSD.

## Figures and Tables

**Table 1:** Very long chain fatty acid analysis in two patients.

	C26:0 (*μ*mol/L)	C24:0 (*μ*mol/L)	C22:0 (*μ*mol/L)	C26:0/C22:0	C24:0/C22:0	Phytanic acid (*μ*mol/L)	Pristanic acid (*μ*mol/L)
Normal	0.6–1.3	37.4–79.4	41.1–90.3	0.011–0.026	0.689–1.008	0.42–3.77	0.0–1.5
Case I	**11.027**	27.041	31.037	**0.355**	0.871	**24.064**	**4.276**
Case II	**10.036**	46.135	29.223	**0.343**	1.579	**21.359**	**3.346**
